# LaCoO_3_ is a promising catalyst for the dry reforming of benzene used as a surrogate of biomass tar

**DOI:** 10.55730/1300-0527.3685

**Published:** 2024-06-15

**Authors:** Başar ÇAĞLAR, Deniz ÜNER

**Affiliations:** 1 Department of Energy Systems Engineering, Faculty of Engineering, İzmir Institute of Technology, İzmir, Turkiye; 2 Department of Chemical Engineering, Faculty of Engineering, Middle East Technical University, Ankara, Turkiye

**Keywords:** Biomass gasification, tar removal, H_2_ production, dry reforming, benzene, LaCoO_3_ catalyst

## Abstract

Tar build-up is one of the bottlenecks of biomass gasification processes. Dry reforming of tar is an alternative solution if the oxygen chemical potential on the catalyst surface is at a sufficient level. For this purpose, an oxygen-donor perovskite, LaCoO_3_, was used as a catalyst for the dry reforming of tar. To circumvent the complexity of the tar and its constituents, the benzene molecule was chosen as a model compound. Dry reforming of benzene vapor on the LaCoO_3_ catalyst was investigated at temperatures of 600, 700, and 800 °C; at CO_2_/C_6_H_6_ ratios of 3, 6, and 12; and at space velocities of 14,000 and 28,000 h^−1^. The conventional Ni(15 wt.%)/Al_2_O_3_ catalyst was also used as a reference material to determine the relative activity of the LaCoO_3_ catalyst. Different characterization techniques such as X-ray diffraction, N_2_ adsorption-desorption, temperature-programmed reduction, and oxidation were used to determine the physicochemical characteristics of the catalysts. The findings demonstrated that the LaCoO_3_ catalyst has higher CO_2_ conversion, higher H_2_ and CO yields, and better stability than the Ni(15 wt.%)/γ-Al_2_O_3_ catalyst. The improvement in activity was attributed to the strong capacity of LaCoO_3_ for oxygen exchange. The transfer of lattice oxygen from the surface of the LaCoO_3_ catalyst facilitates the oxidation of carbon and other surface species and leads to higher conversion and yields.

## 1. Introduction

Biomass is the only renewable source of useful carbon, which can be used directly to replace fossil fuels for fuel and chemical production [1–4]. Among biomass resources, lignocellulosic biomass is the most attractive type of feedstock for producing biofuels and chemicals [5] due to its abundance, low-cost, and nonedible character. For the valorization of lignocellulosic biomass, the conversion of lignin has remained a challenge due to its inactive chemical nature. The lignin content of lignocellulosic biomass varies between 10 and 35 wt.%, which corresponds to up to 40% energy content of the biomass [6]. Therefore, the effective utilization of lignin has great significance for the economic performance of lignocellulosic biomass conversion. Gasification is one of the techniques enabling lignin to be processed along with the other components of lignocellulosic feedstock to produce fuels and chemicals. In gasification, lignocellulosic biomass is converted into a gas mixture at relatively high temperatures (600–1100 °C) in an oxygen-deficient environment. The main drawback of gasification is the undesired tar formation during the process. Tar consists of a mixture of organic compounds [7], including primarily alkylated aromatics, phenolic compounds, and polycyclic aromatic hydrocarbons (PAHs) [8,9]. Tar causes severe problems in downstream applications of biomass gasification processes as it blocks fuel lines and injectors in internal combustion engines, plugs compressors and transfer lines in combined-cycle plants, and poisons fuel cell catalysts (e.g., Pt/C) and Fischer–Tropsch catalysts (e.g., Fe-Co/Al_2_O_3_). In addition to these problems, tar formation leads to decreased efficiency of biomass gasification processes due to incomplete carbon conversion [10]. For the successful development of efficient gasification technology, tar removal or conversion is required.

Catalytic steam and dry reforming are attractive methods used for tar removal since they retain the chemical energies of tar compounds in the product gas, consisting mainly of H_2_ and CO, and provide an opportunity to adjust the composition of the product gas [7]. Although catalytic steam reforming of tar has been addressed more in the literature compared to catalytic dry reforming, the latter has been receiving increased attention recently since it allows the use of CO_2_ as a feedstock. This is important because CO_2_ concentrations in the gasifier outlet (15–20 vol.%) are significantly higher than H_2_O concentrations [11]. Therefore, a dry reforming process also allows both tar removal and CO_2_ valorization.

Tar has a complex chemical nature due to a wide range of chemical compounds (e.g., benzene, naphthalene, acenaphthylene, methylnaphthalene, fluorene, phenanthrene, benzaldehyde, phenols, naphthofurans, benzanthracene, and pyrene). To bypass the complexity of tar and gain further insight into tar decomposition chemistry, various model tar compounds have been extensively studied. Among these studied model tar compounds, toluene, xylene, and styrene were used to represent alkylated aromatics, phenol and cresol were used for phenolic compounds, and benzene and naphthalene were used for aromatic hydrocarbons [12,13]. Among model compounds, benzene has received more attention since it constitutes the largest proportion of tar compositions, specifically for fixed-bed downdraft and fluidized bed gasifiers, while most other compounds are benzene derivatives [14–16]. The benzene ring is a common feature seen in the molecular structure of tar compounds due to its high thermal stability (Table 1) [15,17–19]. Benzene shows higher thermal stability (T_dec_ > 1400 °C) than naphthalene (T_dec_ > 1300 °C) and its other high-molecular-weight derivatives [20]. The composition of benzene and its high thermal stability suggest that benzene decomposition plays a crucial role for the complete conversion of biomass tar. In addition, benzene is a suitable model compound for controllable continuous reactant feeding to a reactor due to its low melting point (i.e., liquid state at room temperature) (Table 1), which is important in regulating reactant flow rate and residence time. For these reasons, benzene was selected as a model tar compound in this study to mimic tar chemistry.

CO_2_ reforming of tar offers the benefit of simultaneous valorization of both compounds. However, the most important problem is catalyst deactivation due to severe carbon build-up. Ni-based catalysts were generally preferred due to the high reforming activity of nickel. Zheng et al. [21] investigated the catalytic dry reforming of toluene on Ni-containing catalysts on different supports such as MgO, *γ*-Al_2_O_3_, *α*-Al_2_O_3_, SiO_2_, and ZrO_2_ at 600 °C at a CO_2_/toluene ratio of 16. They found that the Ni/MgO catalyst exhibited the highest performance with 90% toluene conversion and high stability (5.5% activity loss in 400 min). This was attributed to the strong interactions between Ni and MgO due to the Ni-Mg-O solid solution and the high dispersion of Ni (low particle size of Ni) on the MgO support. Laosiripojana et al. [22] also tested a Ni-based catalyst on different support materials (e.g., MgO-Al_2_O_3_, La_0.8_Ca_0.2_CrO_3_, and La_0.8_Ca_0.2_CrO_3_/MgO-Al_2_O_3_). They used naphthalene as a model compound and analyzed the performance of Ni-Fe bimetallic catalysts. They investigated the effect of catalyst type and CO_2_/naphthalene ratio on the H_2_ yield, determining that H_2_ yield increased with CO_2_/naphthalene ratios between 0.5 and 2 and remained constant above a CO_2_/naphthalene ratio of 2. The highest H_2_ yield was observed as 80% on the Ni-Fe-containing La_0.8_Ca_0.2_CrO_3_/MgO-Al_2_O_3_ catalyst at 700 °C. The dry reforming activities of Ni- and Fe-containing catalysts were also tested by Nam et al. [23]. They investigated the benzene dry reforming activity of SiC-supported Ni and NiFe catalysts in a chemical looping system. They observed that the NiFe/SiC catalyst achieved higher benzene conversion (>90% above 730 °C) and higher H_2_ yield (7.2% at 840 °C), which was linked to higher benzene activation on the Ni surface. In contrast, Abou Rached et al. [24] analyzed the effects of cerium and lanthanum on Ni-Al catalysts for the dry reforming of toluene. They performed experiments at a CO_2_/toluene ratio of 7 between 300 and 800 °C and found that Ce and La promotion did not affect toluene conversion (e.g., 100% above 450 °C), but they improved the stability of the catalyst by decreasing the carbon deposition. Both CeO_2_ and La_2_O_3_ are known for their facile oxygen exchange capacity. For example, La_2_O_3_-based catalysts exhibited superior performance for soot oxidation [25]. On the other hand, CeO_2_ was a favorite candidate for chemical looping processes [26,27]. Hence, both compounds are widely used as oxygen flux agents during catalysis.

Nickel-free catalysts were also tested for the dry reforming of tar compounds. In an early study, Simell et al. [28] evaluated the benzene dry reforming performance of dolomites (CaMg(CO_3_)_2_) at different CO_2_/benzene ratios and temperatures of 750–900 °C. They observed that the benzene conversion varied between 6% and 15% and the H_2_/CO ratio was between 0.1 and 0.25. Another material tested for the dry reforming of tar compounds is olivine (Mg_2_SiO_4_ and Fe_2_SiO_4_). Devi et al. [29] studied the catalytic dry reforming of naphthalene on an olivine catalyst at 900 °C and a CO_2_/naphthalene ratio of approximately 100. They observed 80% naphthalene conversion but did not provide any information on product yields. The dry reforming of tar compounds was also studied on biochar as a catalyst at 800 °C and a CO_2_/toluene ratio of 7 [30]. At the H_2_/CO ratio of 0.4, >95% toluene conversion was observed. It was also found that toluene conversion decreased by 20% after process time of 120 min due to carbon deposition, which blocked the active sites on the biochar. Similar results were obtained in the steam reforming of toluene on the same catalyst.

Several different catalysts were investigated for the dry reforming of tar model compounds under different conditions. Ni-based catalysts show high performance due to their high C-C and C-H bond-breaking activities, although they are prone to coke accumulation on the surface, which induces activity loss in the long run. Similar problems were also observed for non-Ni catalysts. To solve the coke deposition problem, La was employed in the catalyst formulation [24]. Rh-promoted LaCoO_3_ on a γ-Al_2_O_3_ support was found to have high activity for the steam reforming of a biomass–pyrolysis gas mixture as well as high resistance to coke deposition [31–34]. In related studies, the individual effects of Rh and LaCoO_3_ on activity and stability were not clearly identified. Given that process economy is significantly affected by the noble metal content of catalysts, it would be interesting to learn more about the performance of noble metal-free LaCoO_3_ for reforming reactions. In addition, LaCoO_3_ showed high activity towards CO_2_ activation for propane dry reforming [35], reverse water-gas shift reaction [36], and CO_2_ reduction to CO [37] due to its high oxygen exchange capacity. For these reasons, LaCoO_3_ was chosen as a catalyst material in the present study and the catalytic dry reforming of benzene was studied on LaCoO_3_ perovskites, which has not been addressed in the literature to date.

In this study, the dry reforming of benzene on the LaCoO_3_ catalyst was investigated and the activity was compared against a Ni(15 wt.%)/γ-Al_2_O_3_ catalyst, which is a typical tar reforming catalyst formulation used in industrial and academic research [7,38–43]. Both catalysts were characterized by X-ray diffraction (XRD), N_2_ adsorption, and temperature-programmed reduction (TPR) and oxidation (TPO) techniques. Dry reforming experiments were conducted for both catalysts in a tubular quartz reactor and the effects of temperature, CO_2_/C_6_H_6_ ratio, and flow rate were studied. This research will assist in explorations of the potential of LaCoO_3_ as a dry reforming catalyst and the development of new catalyst formulations with high activity, selectivity, and stability to replace conventional Ni/γ-Al_2_O_3_ catalysts.

## 2. Experimental

### 2.1. Catalyst preparation

LaCoO_3_ was prepared by the citrate-based Pechini method due to its advantages over other synthesis methods such as small particle size, high porosity, better control of stoichiometry, homogeneous distribution of ions, relatively low synthesis temperature, and the use of low-cost precursors [44–46]. La_2_O_3_ (99.9%), Co(NO_3_)_2_·6H_2_O (99.9%), citric acid (99.5%), and nitric acid (AR, 60%) were used as starting materials. In the first step, 1.33 g of La_2_O_3_ was dissolved in 5 mL of nitric acid solution and converted to lanthanum nitrate. This nitrate solution was mixed with 2.37 g of cobalt nitrate dissolved in 10 mL of pure water for an atomic ratio of La:Co = 1:1 (LCO). Subsequently, 6.83 g of citric acid was added to this mixture and left to evaporate by stirring for 1 h at 50 °C. At the end of this process, a stable lanthanum cobalt-containing citric acid structure was formed. After 1 h, ethylene glycol was added. The solution was heated to 75 °C with continuous stirring to remove water and accelerate the polyesterification reaction between ethylene glycol and citric acid. After the evaporation of nitrogen-containing gases at this temperature, the remaining material was cooled to room temperature. After this process, a purple viscous solid-liquid mixture was formed. This mixture was turned into a dry gel by heating it to 80 °C in an oven. After drying, the resulting powder was first heated to 350 °C with a heating rate of 10 °C/min and kept at that temperature for 3 h, then heated to 650 °C with a heating rate of 10 °C/min and held for 5 h (Supplementary information, Table S and Figure S1, for an explanation of the two-step calcination). At the end of calcination, LaCoO_3_ nanoparticles were obtained.

In this study, Ni(15 wt.%)/γ-Al_2_O_3_ was used as a reference material to determine the relative activity of LaCoO_3_. The nickel composition was specifically selected considering the composition reported in the literature for commercial catalysts used for tar reforming [7,38–43]. Nickel loading on γ-Al_2_O_3_ material was done by incipient wetness impregnation method as suggested by industrial and academic research [18,19,38]. In this method, 1.48 g of nickel nitrate (Ni(NO_3_)_2_.6H_2_O; Merck, Darmstadt, Germany) was dissolved in an excess amount of pure water (5 mL) and added to γ-Al_2_O_3_ powder (2 g) dropwise under stirring. The resulting mixture was mixed with a magnetic stirrer for 2 h at room temperature. After the mixing process was completed, the material was kept in an oven at 110 °C overnight. Subsequently, the dehydrated materials were left for calcination at 600 °C for 2 h, which allowed the removal of nitrate species (Supplementary information, Figure S2). After calcination, the nitrate-free material was allowed to cool at room temperature and the process was completed.

### 2.2. Catalyst characterization

The crystal structure and size were determined by XRD analysis. XRD spectra were obtained using a Rigaku Ultima Instrument (Rigaku, Tokyo, Japan) with Cu-Kα radiation (λ = 0.154 nm) between 20° and 75° with a scanning rate of 0.1°/s. The average crystalline size was calculated from the Debye–Scherrer formula:


(1) $$L=\frac{0.9\lambda_{K_{\alpha 1}}}{B_{2\theta }\cdot cos\;\theta_{max}}.$$

Here, L is the average crystalline size, *λ_K_*__α_1_ is the wavelength of the X-rays (1.540598 A for Cu-K_a1_ radiation), *B*_2_*_θ_* is the full width at half maximum of the peak, and *θ_max_* is the angle at maximum.

Surface area, pore volume, and average pore size of the catalysts were determined by N_2_ adsorption-desorption at 77 K using a Micromeritics TriStar II instrument (Micromeritics, Norcross, GA, USA). Samples were outgassed at 120 °C for 12 h prior to the experiments.

The reduction and oxidation behaviors of the catalysts were investigated by TPR and TPO experiments in a Micromeritics Chemisorb 2720 instrument. The TPR and TPO experiments were conducted between 25 and 900 °C under the flow of H_2_ in Ar (10% H_2_) and O_2_ in He (2% O_2_) with a heating rate of 5 °C/min. H_2_ and O_2_ consumptions were monitored by a thermal conductivity detector. The quantification of H_2_ consumption was performed using peak areas and the calibration factor determined by the reference Ag_2_O reduction reaction.

### 2.3. Dry reforming tests

**Figure 1:**
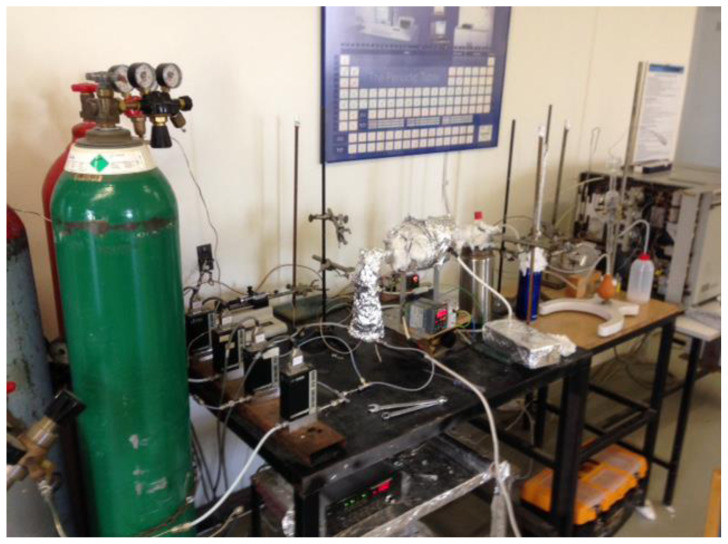
The fixed-bed reaction setup used for dry reforming experiments.

Catalytic tests were performed in a fixed-bed reaction setup as shown in Figure 1. A tubular quartz reactor (ID: 4 mm) placed into a tubular furnace was used as a reactor and the reactor temperature was controlled by a PID controller. For each experiment, 0.1 g of catalyst was loaded into the middle of the reactor with quartz wool before and after the catalytic bed. CO_2_, H_2_ (reducing gas), and N_2_ (inert gas) were fed into the reactor by a mass flow controller (0–200 sccm) while benzene was introduced by a syringe pump. Gas and liquid streams were mixed in a mixer and introduced to the reactor by a heated line to prevent any condensation. Prior to the catalytic tests, catalysts were reduced in the reactor at 600 °C under H_2_ flow (10% H_2_ in N_2_) for 1 h. Catalytic dry reforming tests were first conducted at temperatures of 600, 700, and 800 °C with GHSV of 28,000 h^−1^ (WHSV = 3.5 g benzene/g catalyst per hour) and benzene concentration of 100 g/m^3^ (CO_2_/C_6_H_6_ ratio = 6) for both Ni(15 wt.%)/γ-Al_2_O_3_ and LaCoO_3_ catalysts. The effects of CO_2_/C_6_H_6_ ratio and flow rate were then tested for the LaCoO_3_ catalyst (Table 2). The unreacted benzene at the outlet was collected by a cold trap (i.e. an impinge bottle filled with isopropyl alcohol in a Dewar flask at 0 °C) and the benzene-free gas mixture was sent to a gas chromatography (GC) unit via a 6-way valve with 100-μL sampling port. Gas compositions were analyzed by the GC unit (HP 4890A, Agilent Technologies, Santa Clara, CA, USA) equipped with a TCD detector and Carboxen 1010 capillary column (Sigma Aldrich, St. Louis, MO, USA). For clear separation of the peaks of outlet gases (CO, H_2_, CH_4_, CO_2_, and H_2_O), the GC analysis conditions shown in Table 3 were used.

For each experiment, the percentage H_2_ and CO yields were calculated considering the reaction stoichiometry as follows:


(2) $$Y_{H2}=\frac{{\dot{N}}_{H2}}{3{\dot{N}}_{C6H6,in}}\times 100.$$

(3) $$Y_{CO}=\frac{{\dot{N}}_{CO}}{6{\dot{N}}_{C6H6,in}+{\dot{N}}_{CO2,in}}\times 100.$$

Here, *Ṅ* represents the molar flow rate in mol/min for all considered compounds shown as subscripts with their chemical formulas and the subscript “in” represents the inlet. For all experiments, benzene conversion was assumed to be 100% since no noticeable benzene was detected at the reactor outlet and the high reaction temperature (no kinetic limitation) and high equilibrium constants of benzene reforming and decomposition reactions (Figure S3) favored complete benzene conversion.

## 3. Results and discussion

### 3.1. Thermodynamic analysis of benzene dry reforming

**Figure 2:**
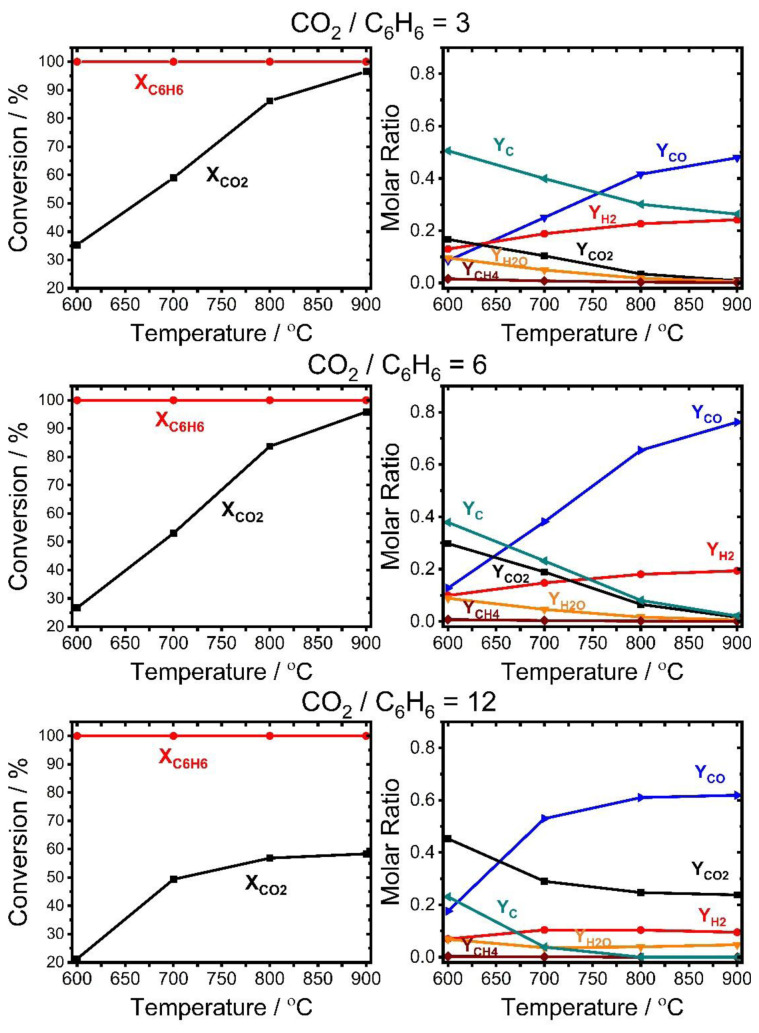
Change of benzene and CO_2_ conversion and product molar fractions at various temperatures and CO_2_/C_6_H_6_ ratios at equilibrium conditions based on Gibbs free energy minimization.

Benzene and CO_2_ conversions and product molar fractions at equilibrium conditions were determined using the Gibbs free energy minimization method to assess the maximum achievable reactant conversions and product yields. Figure 2 shows the change of equilibrium conversion of benzene and CO_2_ and the product molar fractions as a function of temperature between 600 and 900 °C for 3 different CO_2_/C_6_H_6_ molar ratios (3, 6, and 12). As seen from the Figure, benzene was completely converted into products for all CO_2_/benzene ratios and temperatures while there was no complete conversion for CO_2_ even at the substoichiometric condition (CO_2_/C_6_H_6_ = 3) and high temperature. CO_2_ conversion increased with temperature for all CO_2_/C_6_H_6_ ratios due to the endothermic nature of benzene dry reforming reactions (Table 4 and Figure S3). The maximum CO_2_ conversion observed at 900 °C was about 96% for the CO_2_/C_6_H_6_ ratios of 3 and 6, while it was 58% for the CO_2_/C_6_H_6_ ratio of 12 (excess CO_2_ condition). The main equilibrium products were CO, H_2_, carbon, H_2_O, and CH_4_. The change of molar fractions of products including unreacted CO_2_ is also seen in Figure 1. That Figure shows that the amount of CO and H_2_ increased with temperature, whereas the amount of carbon, H_2_O, and CH_4_ decreased along with the unreacted CO_2_. This is mainly associated with endothermic Boudouard, carbon steam reforming, and methane steam reforming reactions (Table 4 and Figure S3). The results indicated that the chemical equilibrium mainly favored CO, H_2_, carbon, and H_2_O formation and that high temperatures are required to maximize CO and H_2_ yield and minimize carbon accumulation.

### 3.2. Catalyst characterization

**Figure 3:**
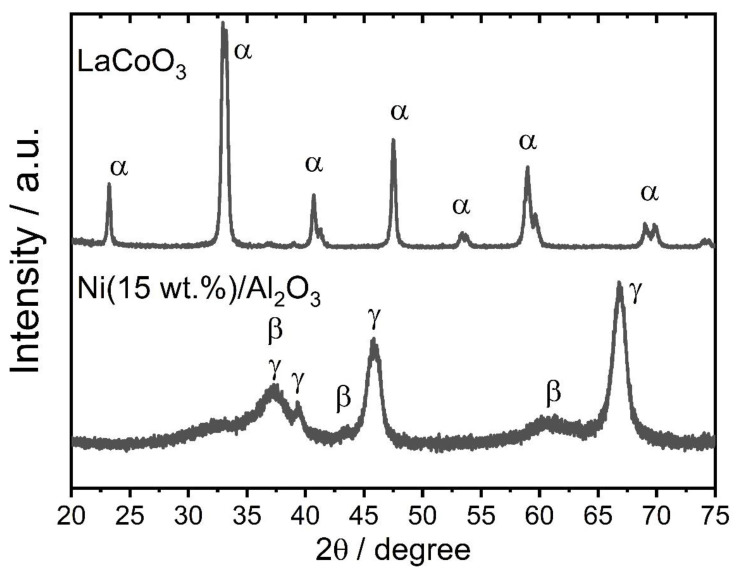
XRD spectra of Ni(15 wt.%)/γ −Al_2_O_3_ and LaCoO_3_ catalysts. α, β, and γ represent LaCoO_3_, NiO, and γ-Al_2_O_3_ phases, respectively.

The crystal structures of both LaCoO_3_ and Ni(15 wt.%)/γ-Al_2_O_3_ catalysts were analyzed by XRD technique. As seen from Figure 3, the XRD spectra of LaCoO_3_ showed the characteristic peaks of hexagonal-rhombohedral LaCoO_3_ (ICDD: 75-0249) at 23.26°, 33.13°, 40.88°, 47.56°, 59.19°, and 69.5°, which were assigned to the (100), (110), (111), (200), (211), and (220) planes. This result confirmed that the synthesis of LaCoO_3_ was successfully done. For Ni(15 wt.%)/γ-Al_2_O_3_ catalyst, the XRD spectra exhibited the presence of γ-Al_2_O_3_ (ICDD: 26-0031) and NiO (ICDD: 47-1049) phases as evidenced by their characteristic peaks. The average crystalline sizes of both LaCoO_3_ and NiO phases for the related catalysts were also determined from the selected planes and peak positions shown in Table 5 using the Debye–Scherrer formula. The average crystalline sizes of LaCoO_3_ and NiO were calculated as 28.8 and 4.4 nm.

The surface area, pore volume, and average pore size of the catalysts were determined using N_2_ adsorption-desorption experiments. As seen from Table 6, the BET surface area and pore volume of the LaCoO_3_ catalyst were almost 7-fold lower than those of the Ni(15 wt.%)/γ-Al_2_O_3_ catalyst while the average pore sizes of both catalysts were similar, which can be explained by the difference in the tortuosity of the catalysts or the difference in the pore shape and structures of the catalysts (Supplementary information, Figure S4).

**Figure 4:**
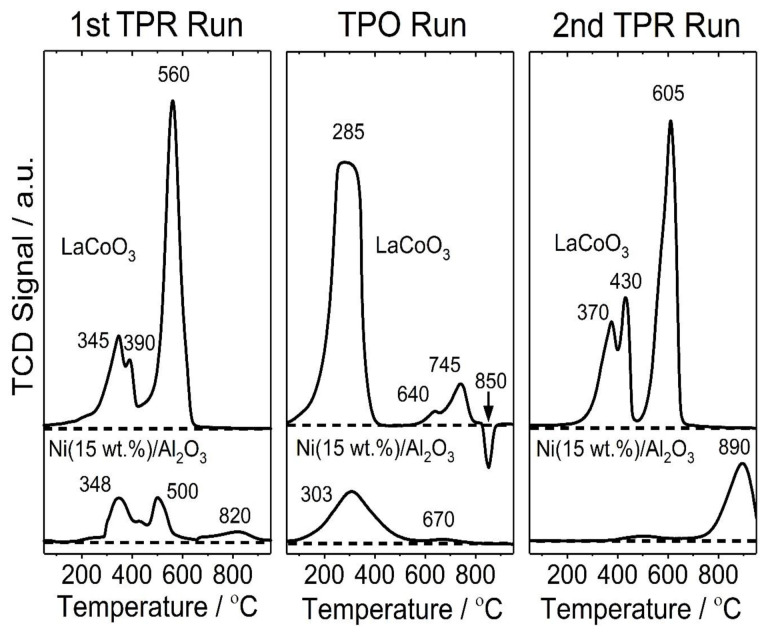
Temperature-programmed reduction and oxidation spectra for Ni(15 wt.%)/γ-Al_2_O_3_ and LaCoO_3_ catalysts. The heating rate for each run is 10 °C/min.

The reduction and oxidation behaviors of the catalysts were investigated by TPR and TPO analyses (Figure 4). The catalysts were first reduced under the flow of H_2_ in Ar (1st TPR), then oxidized under the flow of O_2_ in He (TPO) and successively reduced again (2nd TPR) by a TPR/TPO cycle. The first TPR and TPO tests were used to determine the reduction and oxidation behaviors of the catalysts while the second TPR was used to evaluate the reversibility of the catalysts after oxygen exchange. In the first TPR run, the TPR spectra of the LaCoO_3_ catalyst showed three reduction peaks at 345, 390, and 560 °C. The first two peaks observed at low temperatures were assigned to the reduction of Co^3+^ to Co^2+^ (LaCoO_3_+1/2H_2_→LaCoO_2.5_+1/2H_2_O) while the latter was related to the reduction of Co^2+^ to the metallic cobalt resulting in La_2_O_3_ formation (LaCoO_2.5_+H_2_→1/2La_2_O_3_+Co^0^+H_2_O) [47–50]. The corresponding peak areas of these reduction steps had a 1:1.9 ratio, which is very close to the stoichiometric ratio of 2. The degree of reduction was calculated as 0.75 (Table 7) according to total H_2_ consumption for successive reduction steps, suggesting that only 75% of LaCoO_3_ was accessible for reduction. The TPR spectra of the Ni(15 wt.%)/γ-Al_2_O_3_ catalyst also showed three reduction peaks at 348, 500, and 820 °C with an additional shoulder at a low temperature (435 °C). The peaks at 348 and 500 °C were associated with the reduction of the α-NiO phase and β-NiO phase (NiO+H_2_→Ni+H_2_O), respectively. The former had a weak interaction with the alumina support while the latter had a strong interaction with the support [51–54]. It is known that the TPR peak temperature shifts to higher temperatures with increasing strength between the support and metal, suggesting that the shoulder at 435 °C can be attributed to the reduction of NiO having an intermediate interaction with the support. In contrast to the others, the broad peak at 820 °C was assigned to the reduction of NiAl_2_O_4_ [55–57]. The degree of reduction for the Ni-Al_2_O_3_ catalyst was calculated as 60%, indicating that it has lower reducibility compared to the LaCoO_3_ catalyst.

After the first TPR run the oxidation characteristics of the catalysts were observed by TPO analysis. The TPO spectra of the LaCoO_3_ catalyst showed three oxidation peaks at 285, 640, and 745 °C. The low temperature peak was due to the oxidation of La_2_O_3_ to LaCoO_2.5_ while the high temperature peaks were assigned to the oxidation of LaCoO_2.5_ to LaCoO_3_. A negative peak was also observed in the TPO spectra of the LaCoO_3_ catalyst. This can be attributed to the decomposition of Co_3_O_4_ by oxygen release (Co_3_O_4_ = 3CoO + 1/2O_2_) [58], suggesting that a small amount of Co_3_O_4_ also formed during the oxidation. In contrast to the LaCoO_3_ catalyst, the Ni(15 wt.%)/γ-Al_2_O_3_ catalyst had one strong and one weak oxidation peak at 303 and 670 °C, which were related to the formation of NiAl_2_O_4_ and β-NiO phases [59]. These assignments were also verified by the second TPR run, which showed the reduction peaks of the related phases. The second TPR spectra of the Ni(15 wt.%)/γ-Al_2_O_3_ catalyst showed a significant difference compared to the first one. The peaks associated with the reduction of NiO phases almost disappeared and the intensity of the peak related to the reduction of NiAl_2_O_4_ increased prominently. This indicated that metallic nickel was almost completely inserted in the alumina structure after the TPO run. In contrast to the Ni(15 wt.%)/γ-Al_2_O_3_ catalyst, the reduction peaks in the second TPR run of the LaCoO_3_ catalyst were similar to the ones obtained in the first TPR run with a small shift to high temperatures, suggesting that LaCoO_3_ had good structural stability upon oxygen exchange.

### 3.3. Dry reforming activity

**Figure 5:**
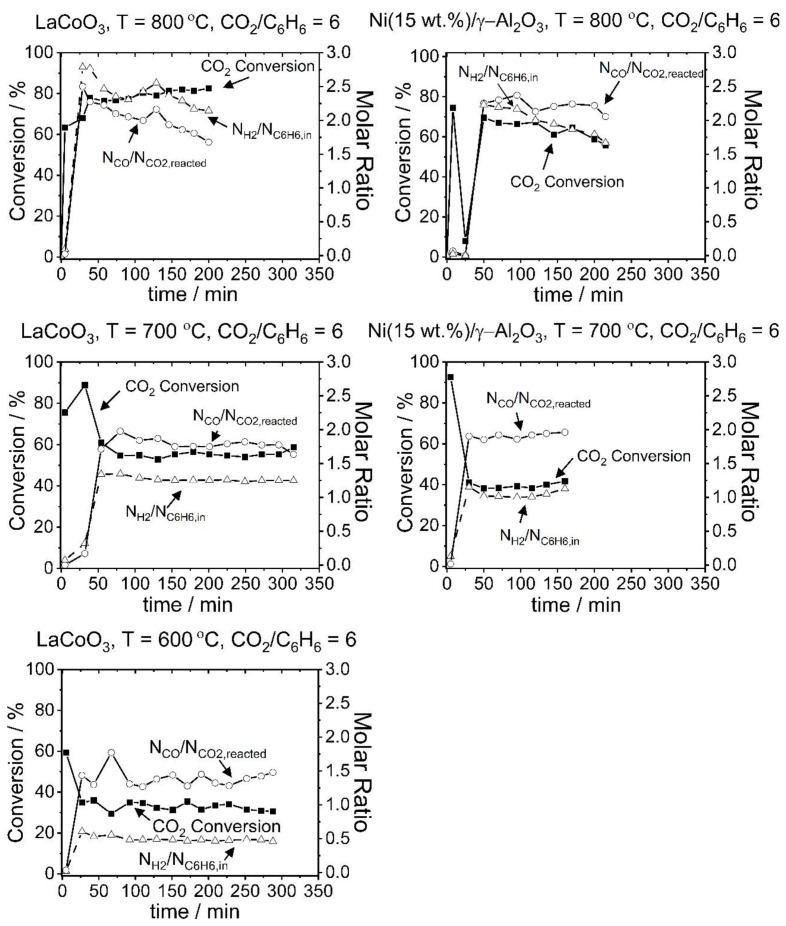
Change of CO_2_ conversion and H_2_/C_6_H_6,in_ and CO/CO_2,reacted_ ratios as a function of time for LaCoO_3_ and Ni(15 wt.%)/γ-Al_2_O_3_ catalysts at 600, 700, and 800 °C and at the CO_2_/C_6_H_6_ ratio of 6.

Dry reforming experiments were performed under the conditions specified in Table 2 for the LaCoO_3_ and Ni(15 wt.%)/γ-Al_2_O_3_ catalysts. Changes of CO_2_ conversion and H_2_/C_6_H_6,in_ and CO/CO_2,reacted_ ratios as a function of time at 600, 700, and 800 °C are shown in Figure 5. As seen from the Figure, a steady response was obtained for all experiments after the first 2 or 3 data points (approximately 50 min). The unsteady behavior in CO_2_ conversion and H_2_/C_6_H_6,in_ and CO/CO_2,reacted_ ratios in the period of interest was attributed to the limited gas passage through the reactor due to pressure build-up, which was removed at the end of this period (Supplementary information, Figure S5). The CO_2_ conversion and H_2_/C_6_H_6,in_ and CO/CO_2,reacted_ ratios obtained at the end of this period (at 50 min) were taken as initial values and average values were calculated based on those initial values and the final values recorded at the end of the experiments. The results are tabulated in Table 8. Equilibrium CO_2_ conversion and H_2_/C_6_H_6,in_ and CO/CO_2,reacted_ ratios were also found based on Gibbs free energy minimization (see subsection 3.1 and Figure S6) and are listed in Table 9 to allow an evaluation of the results in comparison to equilibrium conditions. The reaction results indicated that at both 700 and 800 °C the LaCoO_3_ catalyst had a higher CO_2_ conversion and H_2_/C_6_H_6,in_ ratio than the Ni(15 wt.%)/γ-Al_2_O_3_ catalyst, while it had a lower CO/CO_2,reacted_ ratio compared to its counterpart. At 800 °C the equilibrium CO_2_ conversion was almost achieved (80.1%) for the LaCoO_3_ catalyst while the CO_2_ conversion of the Ni(15 wt.%)/γ-Al_2_O_3_ catalyst was 62.7%. At 800 °C the H_2_/C_6_H_6,in_ and CO/CO_2,reacted_ ratios observed for the LaCoO_3_ catalyst at the end of the experiment were 2.45 and 1.97, respectively. The corresponding H_2_ and CO yields were 81.8% and 78.4%, respectively, indicating that benzene dry reforming primarily results in H_2_ and CO formation. The H_2_ and CO yields for the Ni(15 wt.%)/γ-Al_2_O_3_ catalyst were 65.0 and 67.9, respectively, suggesting that the LaCoO_3_ catalyst outperformed the Ni(15 wt.%)/γ-Al_2_O_3_ catalyst in terms of H_2_ and CO production. For both catalysts, the CO_2_ conversion and H_2_/C_6_H_6,in_ and CO/CO_2,reacted_ ratios obtained at 800 °C showed a steady decline associated with gas disruption due to carbon accumulation in the reactor. The carbon formation on the catalyst surface was confirmed by TGA analysis of the used catalysts (Supplementary information, Figures S7–S9). The gas disruption due to carbon accumulation was less prominent for the results obtained at 700 and 600 °C, but it still caused a small deviation from the steady response due to the resulting uneven gas distribution at the sampling port. This may explain why the average CO_2_ conversions and CO yields of the LaCoO_3_ catalyst were higher than the related equilibrium conversions and yields at 700 and 600 °C (Table 9) to an extent beyond the specified uncertainties (e.g., at 600 °C 32.8 vs. 26.7, with ±5% uncertainty), which only accounted for instrumental and calibration errors. Although the relevant data are beyond the error margin by a small difference, it is still possible to infer that the LaCoO_3_ catalyst has CO_2_ conversion and CO yield very close to equilibrium values and higher than those for the Ni(15 wt.%)/γ-Al_2_O_3_ catalyst. The effects of temperature on CO_2_ conversion and CO and H_2_ yields were also clearly seen in the results. There was a significant decrease in conversion and yields with decreasing temperature for both catalysts. This was attributed to the strong endothermicity of dry reforming reactions (Table 4 and Figure S3).

One of the important considerations regarding the results is the difference in the experimental time for each test. This is related to the reactor blockage caused by carbon build-up. When the gas flow was significantly disrupted, the operation was discontinued. For that reason, no reliable data were obtained for the Ni(15 wt.%)/γ-Al_2_O_3_ catalyst at 600 °C and such data are not presented here. The absence of experimental data at 600 °C and a lower experimental time (160 min) at 700 °C for the Ni(15 wt.%)/γ-Al_2_O_3_ catalyst in comparison to the LaCoO_3_ catalyst (288 min for 600 °C and 315 min for 700 °C) suggested that the LaCoO_3_ catalyst showed higher stability than the Ni(15 wt.%)/γ-Al_2_O_3_ catalyst.

**Figure 6:**
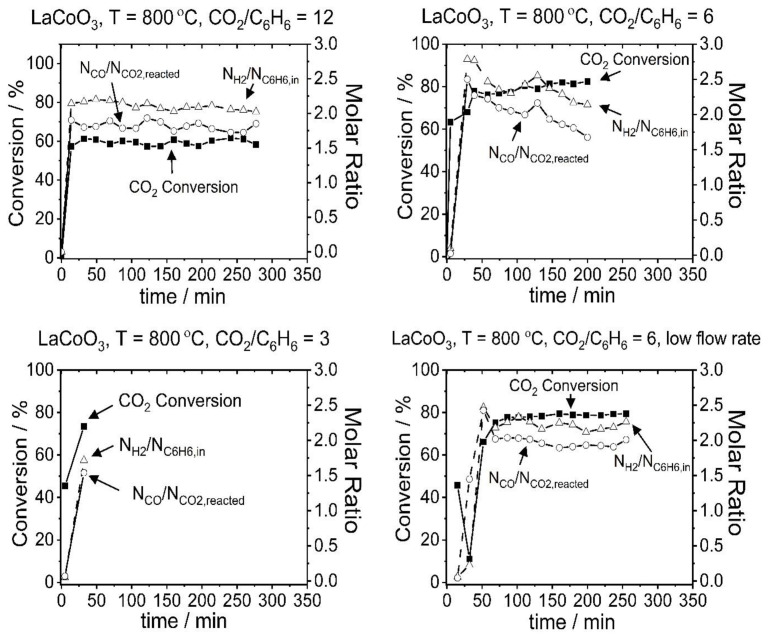
CO_2_ conversion and H_2_/C_6_H_6,in_ and CO/CO_2,reacted_ ratios obtained for the LaCoO_3_ catalyst at 800 °C and at the CO_2_/C_6_H_6_ ratio of 12 (top left), 6 (top right), and 3 (bottom left) and at a low flow rate (bottom right).

The effects of CO_2_/C_6_H_6_ ratio and flow rate on conversion and yields were also studied for the LaCoO_3_ catalyst. Figure 6 shows the change of CO_2_ conversion values and H_2_/C_6_H_6,in_ and CO/CO_2,reacted_ ratios as a function of time at 800 °C and three different CO_2_/C_6_H_6_ ratios (3, 6, and 12). As seen from the Figure, a lower CO_2_ conversion and H_2_/C_6_H_6,in_ ratio were observed at the CO_2_/C_6_H_6_ ratio of 12 compared to the experiment conducted at CO_2_/C_6_H_6_ of 6, whereas CO_in_/CO_2,reacted_ remained almost constant on average. The relatively low CO_2_ conversion can be explained by the presence of excess CO_2_ while the low H_2,in_/C_6_H_6,in_ ratio and the similar CO_in_/CO_2,reacted_ values were attributed to the differences in the reaction occurring under related conditions. At the CO_2_/C_6_H_6_ ratio of 6, stoichiometric benzene dry reforming (BDR-1 in Table 4) mainly occurred, whereas with excess CO_2_ a dry reforming reaction with a stoichiometry ratio of 9 CO_2_/C_6_H_6_ (BDR-2 in Table 4) and carbon steam reforming reaction (CSR in Table 4) occurred as well. The dry reforming reaction with a stoichiometry ratio of 9 led to a decrease in H_2_ yield while the carbon steam reforming reaction promoted CO formation and maintained the CO yield. A lower CO_2_ conversion and H_2_ yield were also observed under substoichiometric conditions (CO_2_/C_6_H_6_ = 3), accompanied by a lower CO yield. This was attributed to carbon deposition. For this reason, a single data point was obtained for this condition. The effect of flow rate was also tested under stoichiometric conditions by decreasing the flow rate by 2-fold. No prominent change was observed compared to the high flow rate condition, suggesting that external mass transfer resistance was not effective in the relevant flow rate range (14,000–28,000 h^−1^).

**Figure 7:**
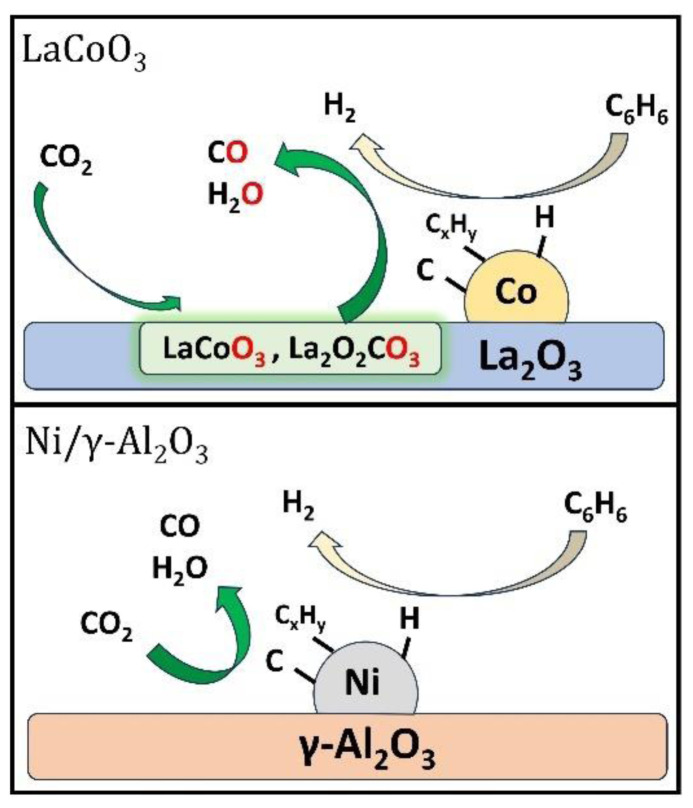
Presumed reaction schemes for benzene dry reforming on the LaCoO_3_ (top) and Ni/γ-Al_2_O_3_ (bottom) catalysts.

The dry reforming results showed that the LaCoO_3_ catalyst had higher CO_2_ conversion and CO and H_2_ yields compared to the reference Ni(15 wt.%)/γ-Al_2_O_3_ catalyst. This could be linked to the high oxygen exchange capacity of the LaCoO_3_ catalyst. Prior to the dry reforming experiment, catalyst reduction at 600 °C under H_2_ environment resulted in Co and La_2_O_3_ formation for the LaCoO_3_ catalyst, while it yielded Ni and Al_2_O_3_ for the Ni(15 wt.%)/γ-Al_2_O_3_ catalyst as seen from the TPR analysis. It is known from the literature that benzene adsorbs on metal surfaces via its two atoms, and it subsequently decomposes to C_x_H_y_, carbon, and H_2_ via C-C and C-H bond scissions [28,60], which occurs on metal sites of both catalyst surfaces (Rxn 1–3 in Table 10). However, the catalysts differed from each other in terms of the reactions between CO_2_ and catalyst surfaces. On the LaCoO_3_ surface, Co and La_2_O_3_ reacted with CO_2_ to form CO and LaCoO_3_ (Rxn 4, Table 10) [35,37,61] or La_2_O_3_ reacted with CO_2_ to form La_2_O_2_CO_3_ (Rxn 5, Table 10) [62,63] with the exchange of the lattice oxygen. The resulting species further reacted with C_x_H_y_ species, surface hydrogen, and surface carbon to produce CO, H_2_, and H_2_O (Rxn 6–11). These reaction steps were less likely to happen on the Ni(15 wt.%)/γ-Al_2_O_3_ surface, where CO_2_ directly reacted with surface moieties (C_x_H_y_ species, surface carbon) to produce CO and H_2_O (Rxn 12–13). The faster kinetics of the surface CO_2_ reaction and the following oxidation of surface species on the LaCoO_3_ catalyst led to higher CO_2_ conversion and product yields compared to its counterpart due to its high oxygen exchange capability as seen from TPR analysis (see subsection 3.2). The presumed reaction schemes for both catalysts are shown in Figure 7.

The performance of the LaCoO_3_ was evaluated in comparison to the conventional Ni-Al_2_O_3_ catalyst for benzene dry reforming, which mimics catalytic tar conversion. The results indicated that the LaCoO_3_ catalyst outperformed the Ni-Al_2_O_3_ catalyst in terms of CO_2_ conversion and CO and H_2_ yields. For all temperatures studied (600, 700, and 800 °C), the LaCoO_3_ catalyst showed about 18% greater CO_2_ conversion and approximately 5%–17% higher H_2_ and CO yields than the Ni-Al_2_O_3_ catalyst. The benzene dry reforming activity of the LaCoO_3_ catalyst was also found to be better than that of other catalysts tested in the literature, such as dolomite (CaO.MgO) [28] and NiFe/SiC [23]. For the dolomite catalyst, 33% benzene conversion was reported at 800 °C and stoichiometric CO_2_ conditions, which is much lower than the benzene conversion for the LaCoO_3_ catalyst in the present study under similar conditions (800 °C, CO_2_/C_6_H_6_ = 12) considering that the H_2_ yield was 67.5% (corresponding to at least 67.5% benzene conversion if benzene is assumed to convert only to H_2_). Similarly, the H_2_ and CO yields observed on the NiFe/SiC catalyst at 840 °C under excess CO_2_ were 7.2% and 20%, respectively. Under similar conditions (800 °C, CO_2_/C_6_H_6_ = 12), the H_2_ and CO yields were 69.9% and 72.7% for the LaCoO_3_ catalyst. These findings suggest that the LaCoO_3_ catalyst is a promising catalyst candidate for catalytic tar removal and CO_2_ valorization as evidenced by its relatively high activity towards benzene dry reforming.

## 4. Conclusion

Benzene dry reforming provides valuable information for the catalytic cracking of tar used as a posttreatment unit in biomass gasification. In this study, benzene was taken as a model molecule for tar compounds due to its high thermal stability and abundance at the outlet of biomass gasifiers while LaCoO_3_ was selected as the dry reforming catalyst to overcome the challenges observed for conventional Ni-Al_2_O_3_ catalysts (e.g., coke deposition, low stability). Catalyst characterization was performed to determine the crystal structure, surface area, and reduction/oxidation behavior of the catalyst, which helped in determining the structure–activity relationship. Dry reforming activity tests of the LaCoO_3_ catalyst were conducted at three different temperatures (600, 700, and 800 °C) and CO_2_/C_6_H_6_ ratios (3, 6, and 12) and two different space velocities (14,000 and 28,000 h^−1^) and compared to the Ni(15 wt.%)/γ-Al_2_O_3_ catalyst. The results showed that the LaCoO_3_ catalyst achieved 18% higher CO_2_ conversion, 5%–17% higher H_2_ yield, 10% higher CO yield, and better stability than the Ni(15 wt.%)/γ-Al_2_O_3_ catalyst. The difference in reactivity was linked to the high oxygen exchange ability of the LaCoO_3_ catalyst, which promotes the oxidation of carbon and other surface species by lattice oxygen transfer, leading to higher CO_2_ conversion and CO and H_2_ yields.

## Supplementary information

### S1. The reasoning behind the two-step calcination used for the preparation of LaCoO_3_ via sol-gel based Pechini method

In the preparation of LaCoO3 by citrate-based Pechini method, a two-step calcination was used to prevent a significant reduction of surface area and pore volume of the sample, which was observed when the sample was directly heated to 650 °C (Table S).

**Table S:** The comparison of surface area and pore volume obtained from two-stage heating and direct ramp up during calcination.

Method	BET surface area (m ^2^/g)	Pore volume (cm ^3^/g)
Two-stage calcination	5.91	0.005
Direct ramp up	0.15	0.001

The reason for the reduction in the surface area and pore volume was attributed to the pore collapse due to the fast decomposition of lanthanum and cobalt nitrates as evidenced by the weight loss observed at around 350 °C in TGA analysis (Figure S1). Due to this reason, we first heated the sample to the nitrate decomposition temperature 350 °C and waited at the related temperature for 3 h to slow down nitrate decomposition rate and to prevent the resulting pore collapse.

Figure S1 The TGA curve of the LaCoO3 prepared by citrate-based Pechini method before calcination.

### S2. The reasoning behind the selection of calcination temperature for the Ni(15 wt.%)/γ-Al_2_O_3_ catalyst prepared by incipient wetness impregnation method

The Ni(15 wt.%)/γ-Al_2_O_3_ catalyst was prepared by incipient wetness impregnation method, which is commonly used technique for the preparation of catalysts due to technical simplicity, low costs and limited amount of waste. The method mainly consists of impregnation, drying, and calcination steps. The calcination step has a dramatic effect on the impurities sourced from precursors and crystal structure of catalysts. In the current study, TGA analysis was performed to determine a suitable calcination temperature for the Ni(15 wt.%)/γ-Al_2_O_3_ catalyst (Figure S2). TGA analysis shows that all nitrates are removed above 500 °C, as evidenced by no weight loss above this temperature interval.

Figure S2 The TGA curve of the Ni(15 wt.%)/γ-Al2O3 catalyst prepared by incipient wetness impregnation method before calcination.

### S3. The change of equilibrium constants of dry reforming reactions as a function of temperature

Figure S3 The change of equilibrium constants of reactions occurring at dry reforming conditions as a function of temperature.

### S4. N_2_ adsorption-desorption isotherms of LaCoO_3_ and Ni/Al_2_O_3_

N_2_ adsorption-desorption experiments show that the BET surface area and pore volume of the LaCoO_3_ catalyst are almost 7-fold lower than those of the Ni(15 wt.%)/γ-Al_2_O_3_ catalyst while the average pore sizes of both catalysts are similar. The reason for the similarity in average pore diameters of catalysts while the difference in pore volume and surface area can be explained by the difference in tortuosity of the catalysts or the difference in their pore structure and shapes. The latter was analyzed by N_2_ adsorption-desorption isotherms as seen in Figure S4. The Figure shows that the LaCoO_3_ catalyst exhibits Type IV isotherm H4 hysteresis, which is an indication of narrow slit-like pore, while the Ni/Al_2_O_3_ catalyst shows Type IV isotherm H2 hysteresis suggesting the presence of ink bottle shape pores. Since the average pore size in the current study was determined based on the BJH method assuming that all pores open-ended cylindrical, this may also lead to similar average pore size, but different pore volumes.

Figure S4 N_2_ adsorption-desorption isotherms of LaCoO_3_ (top) and Ni/Al_2_O_3_ (bottom).

### S5. The reasoning for the abnormality of conversion and yields in the first 50 min

CO_2_ conversion and H_2_ and CO yields change significantly with the first 50 min compared to those obtained after 50 min (Figures 4 and 5). The reason for this abrupt change in conversion and yields are related to the reaction setup used in this study. For all experiments, the position of powder catalyst was fixed tightly in the middle of reactor by quartz wool on both side of the catalyst bed to prevent any uncontrolled gas passage (e.g., free gas flow next to the reactor wall without contacting catalytic bed) through the reactor. This leads to pressure build-up in the first 50 min and limits gas flow at the reactor outlet, which is clearly seen in the gas flow rate measurement at the reactor outlet (Figure S5). When the pressure reaches a certain value, the gas blockage is removed, and the inlet and outlet gas flow rate are equalized. Since the outlet molar flow rate of each gas is calculated by the product of total gas flow rate and gas composition (determined by GC), the CO_2_ conversion was found to be higher while H_2_ and CO yields were determined to be lower than those observed after gas flow rate stabilization (i.e. after 50 min).

Figure S5 The variation of gas flow rate as function of time for the LaCoO_3_ catalyst at the conditions of 800 °C and the CO_2_/C_6_H_6_ ratio of 6.

### S6. Equilibrium conversion and yields at different reactant ratios and temperatures

Figure S6 Equilibrium conversion of CO_2_ and H_2_ and CO yields (left panel) along with H_2_/C_6_H_6,in_ and CO/CO_2,reacted_ ratios at equilibrium conditions (right panel) determined based on Gibbs free energy minimization.

### S7. Postreaction characterization by TGA for confirmation of carbon formation

At the end of the dry reforming experiment, the used catalysts were collected from the reactor along with quartz wool and used for TGA analysis. Since it is difficult to separate the used catalyst from the quartz wool, the quartz wool was also included in the sample for TGA analysis. TGA analyses were performed between 25 and 900 °C with a heating rate of 10 °C/min under air atmosphere. TGA curves of the used LaCoO_3_ and Ni/γ-Al_2_O_3_ catalyst obtained after the dry reforming experiment at 800 °C and at the CO_2_/C_6_H_6_ ratio of 6 are shown in Figures S7 and S8.

Figures show a typical weight loss between 500 and 700 °C for both catalysts, which is related to carbon oxidation. This clearly proves the carbon accumulation on the surface of catalysts during the dry reforming reaction. The total amounts of carbon accumulated on both catalyst surfaces were also calculated. The results show that carbon formation rate (determined by the total amount of carbon divided by the reaction duration) is higher on the Ni/γ-Al_2_O_3_ catalyst compared to that on LaCoO_3_ catalyst, but this is not a reasonable comparison of the instant carbon yield of the catalysts since it is difficult to determine the exact reaction duration leading to carbon formation. When carbon builds up on the surface, it starts to block gas passage. The gas flow rate decreases slowly until the moment where an almost complete blockage occurs. Therefore, the averaging the carbon formation in time does not give correct assessment due to instabilities in the system.

Figure S7 TGA analysis of the used LaCoO_3_ catalyst obtained after the dry reforming experiment at 800 °C and at the CO_2_/C_6_H_6_ ratio of 6.

Figure S8 TGA analysis of the used Ni/γ-Al_2_O_3_ catalyst obtained after the dry reforming experiment at 800°C and at the CO_2_/C_6_H_6_ ratio of 6.

The TGA analysis was also performed for the used LaCoO_3_ and Ni/γ-Al_2_O_3_ catalysts after the dry reforming experiment at 600 and 700 °C at the CO_2_/C_6_H_6_ ratio of 6. As seen from Figure S9, the TGA curve of the used LaCoO_3_ catalyst presents two weight increases at 300 and 395 °C. This is attributed to the oxidation of La_2_O_3_ to LaCoO_2.5_ as seen in the TPO analysis (Figure 4). The related weight increase indicates that a part of the catalyst remains in the reduced form (i.e. Co and La_2_O_3_) during dry reforming suggesting that a part of oxygen is exchanged to the surface for carbon oxidation. Different from the LaCoO_3_ catalyst, the related oxidation peaks are not present in the TGA curve of the Ni/γ-Al_2_O_3_ catalyst. This explains the higher CO yields observed on the LaCoO_3_ catalyst compared to that of the Ni/γ-Al_2_O_3_ catalyst. The TGA curves of both catalysts indicate weight decrease above 500 °C, which is related to oxidation of surface carbon. The relative weight decreases with respect to the initial weight before the carbon oxidation shows that the weight percentages of carbon in the used catalysts are 7.8% and 14.0% for LaCoO_3_ and Ni/γ-Al_2_O_3_ catalysts, respectively. This suggests that the LaCoO_3_ catalyst is less prone to coke formation compared to the Ni/γ-Al_2_O_3_ catalyst due to its high oxygen exchange capacity.

Figure S9 TGA analysis of the used LaCoO_3_ (left panel) and Ni/γ-Al_2_O_3_ (right panel) catalysts obtained after the dry reforming experiment at 700 °C and at the CO_2_/C_6_H_6_ ratio of 6.

## Figures and Tables

**Table 1:** Typical tar compounds observed at gasification outlet.

Chemical compound	Chemical structure	Boiling point (°C)	Melting point (°C)
Benzene	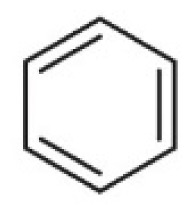	80.1	5.53
Naphthalene	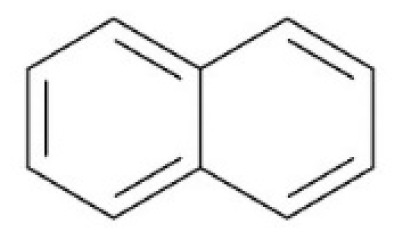	218.0	78.2
Acenaphthylene	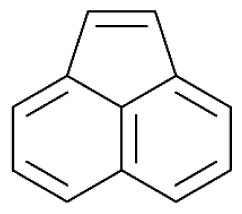	280.0	91.8
Methylnaphthalene	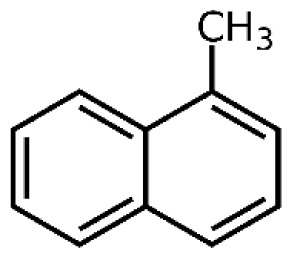	240.0	−22.0
Fluorene	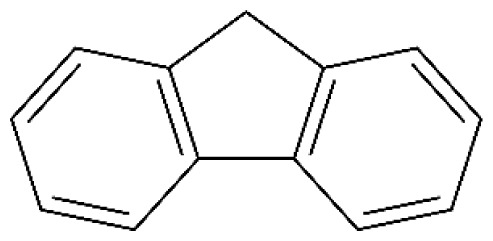	295.0	116.0
Phenanthrene	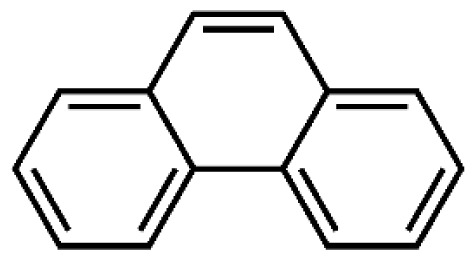	332.0	101
Benzaldehyde	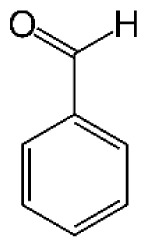	178.1	−57.0
Phenol	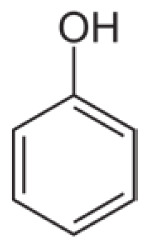	181.7	40.5
Benzanthracene	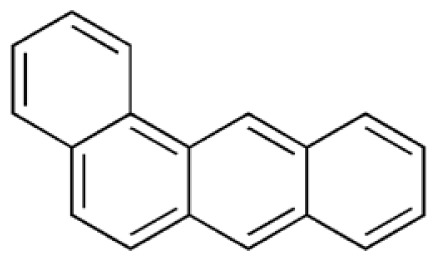	438.0	158

**Table 2:** Dry reforming test conditions.

Catalysts	Temperature (°C)	CO_2_/C_6_H_6_ ratio	GHSV (h^−1^)	WHSV (g benzene/g catalyst per hour)	Benzene concentration (g/m^3^)	Residence time (s)
LaCoO_3_, Ni(15 wt.%)-Al_2_O_3_	600, 700, 800	6	28,000	3.5	100	0.13
LaCoO_3_	800	12	28,000	3.5	100	0.13
LaCoO_3_	800	3	28,000	3.5	100	0.13
LaCoO_3_	800	6	14,000	1.75	100	0.26

**Table 3:** GC analysis conditions.

Column	Oven temperature	Injection temperature (°C)	Detector temperature (°C)	Amount of gas injection (μL)
Carboxen 1010	35 °C (held for 5 min), heated to 250 °C with a rate of 24 °C/min	200	230	100

**Table 4:** Possible reactions occurring in the reaction medium.

No	Reaction type	Chemical equation	Δ*ĥ_rxn_*(kJ/mol)
**1**	Boudouard	C + CO_2_ ↔ CO	172.4
**2**	Carbon steam reforming (CSR)	C + H_2_O (g) ↔ CO + H_2_	131.3
**3**	Water-gas shift (WGS)	CO + H_2_O (g) ↔ CO_2_ + H_2_	−41.1
**4**	Benzene dry reforming (BDR-1)	C_6_H_6_ + 6CO_2_ ↔ 12CO + 3H_2_	951.6
**5**	Benzene dry reforming (BDR-2)	C_6_H_6_ + 9CO_2_ ↔ 9CO + 3H_2_O(g)	1075.0
**6**	Benzene decomposition (BD)	C_6_H_6_ ↔ 6C + 3H_2_	−82.9
**7**	Methane steam reforming (MSR)	CH_4_ + H_2_O (g) ↔ CO + 3H_2_	206.1
**8**	Methane dry reforming	CH_4_ + CO_2_ ↔ 2CO + 2H_2_	247.0
**9**	Methanation	C + H_2_ ↔ CH_4_	−75.0

**Table 5:** Average crystalline sizes of catalysts obtained from XRD spectra.

Catalyst - phase	Facet	Peak position (2q)	Average crystalline size (nm)
Ni(15 wt.%)/γ-Al_2_O_3_ - NiO	111	37.26	4.4
LaCoO_3_	110	33.13	28.8

**Table 6:** BET surface area, pore volume, and average pore size of catalysts obtained from N_2_ adsorption and desorption experiments.

Catalyst	BET surface area (m^2^/g)	Pore volume (cm^3^/g)	Average pore size (nm)
Ni(15 wt.%)/γ-Al_2_O_3_	43.58	0.035	4.32
LaCoO_3_	5.91	0.005	4.33

**Table 7:** H_2_ consumption of catalysts and their degree of reduction obtained from TPR runs.

Catalysts	H_2_ consumption (mmol/g)			Degree of reduction	

	1st TPR	2nd TPR*	Theoretical**	1st TPR	2nd TPR
	
Ni-Al_2_O_3_	1.32	1.47	2.22	0.59	0.66
LaCoO_3_	4.56	4.71	6.10	0.75	0.77

* The weight of the catalyst at the beginning of the first TPR run was taken for the calculation.

** The theoretical H_2_ consumptions for the Ni-Al_2_O_3_ and LaCoO_3_ catalysts were determined based on the stoichiometry of the following reactions: (i) NiO + Al_2_O_3_ + H_2_ = Ni + Al_2_O_3_ + H_2_O; (ii) LaCoO_3_ +3/2 H_2_ = 1/2 La_2_O_3_ + Co + 3/2 H_2_O.

**Table 8:** Average values of CO_2_ conversion, H_2_ and CO yields, and H_2_/C_6_H_6,in_ and CO/CO_2,reacted_ ratios obtained for LaCoO_3_ and Ni(15 wt.%)/γ-Al_2_O_3_ catalysts at different temperatures, N_CO2_/N_C6H6_ ratios, and GHSV (h^−1^). The uncertainties of conversion and yield values are ±5% while the uncertainties of ratios are ±0.2.

Reaction conditions	LaCoO_3_					Ni(15 wt.%)/γ-Al_2_O_3_				
	X_CO2_	N_H2_/N_C6H6_	Y_H2_ (%)	N_CO_/N_CO2_	Y_CO_	X_CO2_	N_H2_/N_C6H6_	Y_H2_ (%)	N_CO_/N_CO2_	Y_CO_ (%)
600 °C, N_CO2_/N_C6H6_ = 6, 28,000 h^−1^	32.8	0.53	17.8	1.46	23.7	NA				
700 °C, N_CO2_/N_C6H6_ = 6, 28,000 h^−1^	59.8	1.29	43.2	1.67	49.8	41.3	1.14	38.0	1.93	40.0
800 °C, N_CO2_/N_C6H6_ = 6, 28,000 h^−1^	80.1	2.45	81.8	1.97	78.4	62.7	1.95	65.0	2.15	67.9
800 °C, N_CO2_/N_C6H6_ = 12, 28,000 h^−1^	59.8	2.09	69.9	1.82	72.7	NA				
800 °C, N_CO2_/N_C6H6_ = 3, 28,000 h^−1^	73.5	1.72	57.2	1.54	38.7	NA				
800 °C, N_CO2_/N_C6H6_ = 6, 14,000 h^−1^	72.7	2.37	78.9	2.21	78.1	NA				

**Table 9:** Equilibrium CO_2_ conversion, H_2_ and CO yields, and H_2,in_/C_6_H_6,in_ and CO_in_/CO_2,reacted_ ratios at different temperatures and N_CO2_/N_C6H6_ ratios.

Reaction conditions	X_CO2_	N_H2_/N_C6H6_	Y_H2_ (%)	N_CO_/N_CO2_	Y_CO_ (%)
600 °C, N_CO2_/N_C6H6_ = 6	26.7	1.47	49.0	1.18	15.7
700 °C, N_CO2_/N_C6H6_ = 6	53.0	2.20	73.3	1.79	47.3
800 °C, N_CO2_/N_C6H6_ = 6	83.7	2.70	90.0	1.95	81.6
800 °C, N_CO2_/N_C6H6_ = 12	56.8	2.17	72.3	1.88	71.1
800 °C, N_CO2_/N_C6H6_ = 3	86.2	2.70	90.0	1.92	55.1

**Table 10:** Presumed reaction steps occurring on catalyst surfaces.

No.	Reaction*	Catalysts
1	C_6_H_6_ + M = M-C_6_H_6_	LaCoO_3_, Ni(15 wt.%)/γ-Al_2_O_3_ M: Co, Ni
2	M-C_6_H_6_ = M-C_x_H_y_ + M-zC + (6-y)/2 H_2_	
3	M-C_x_H_y_ = M-xC + y/2 H_2_ (or +M-yH)	
4	3/2 CO_2_ + 1/2 La_2_O_3_ + Co = 3/2 CO + LaCoO_3_	LaCoO_3_
5	CO_2_ + La_2_O_3_ = La_2_O_2_CO_3_	
6	LaCoO_3_ + Co-C_x_H_y_ = zCO + y/2 H_2_ + 1/2 La_2_O_3_ + 2Co	
7	LaCoO_3_ + 3/2 Co-C = 3/2 CO + 1/2 La_2_O_3_ + Co	
8	LaCoO_3_ + 3 Co-H = 3/2 H_2_O + 1/2 La_2_O_3_ + Co	
9	La_2_O_2_CO_3_ + Co-C_x_H_y_ = zCO + y/2 H_2_ + La_2_O_3_	
10	La_2_O_2_CO_3_ + Co-C = 2CO + La_2_O_3_	
11	La_2_O_2_CO_3_ + 2Co-H = H_2_O + CO + La_2_O_3_	
12	Ni-C_x_H_y_+ Al_2_O_3_-CO_2_ = (2-y)/2CO + y/2 H_2_O + Ni + Al_2_O_3_	Ni(15 wt.%)/γ-Al_2_O_3_
13	Ni-C + Al_2_O_3_-CO_2_ = 2CO + Ni + Al_2_O_3_	

* x, y, and z represent stoichiometric coefficients.
