# Supplementary figures and images for: LaCoO3 is a promising catalyst for the dry reforming of benzene used as a surrogate of biomass tar
Source: Turk J Chem. 2024 Jun 15;48(4):643–58. doi: 10.55730/1300-0527.3685 (PMC11407365; doi:10.55730/1300-0527.3685)

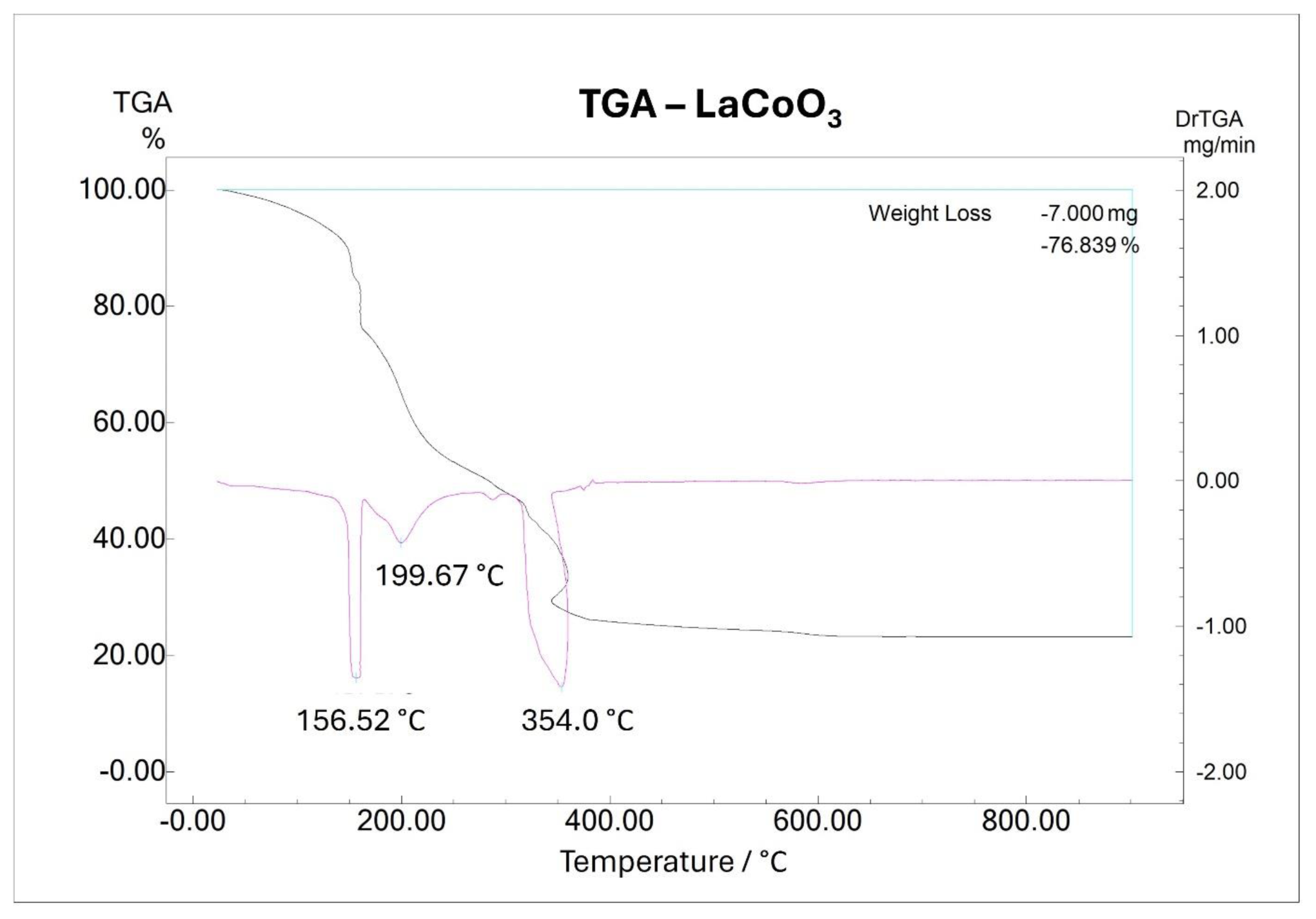

Supplement: Figure S1 — The TGA curve of the LaCoO3 prepared by citrate-based Pechini method before calcination. [file tjc-48-04-643s1.tif]

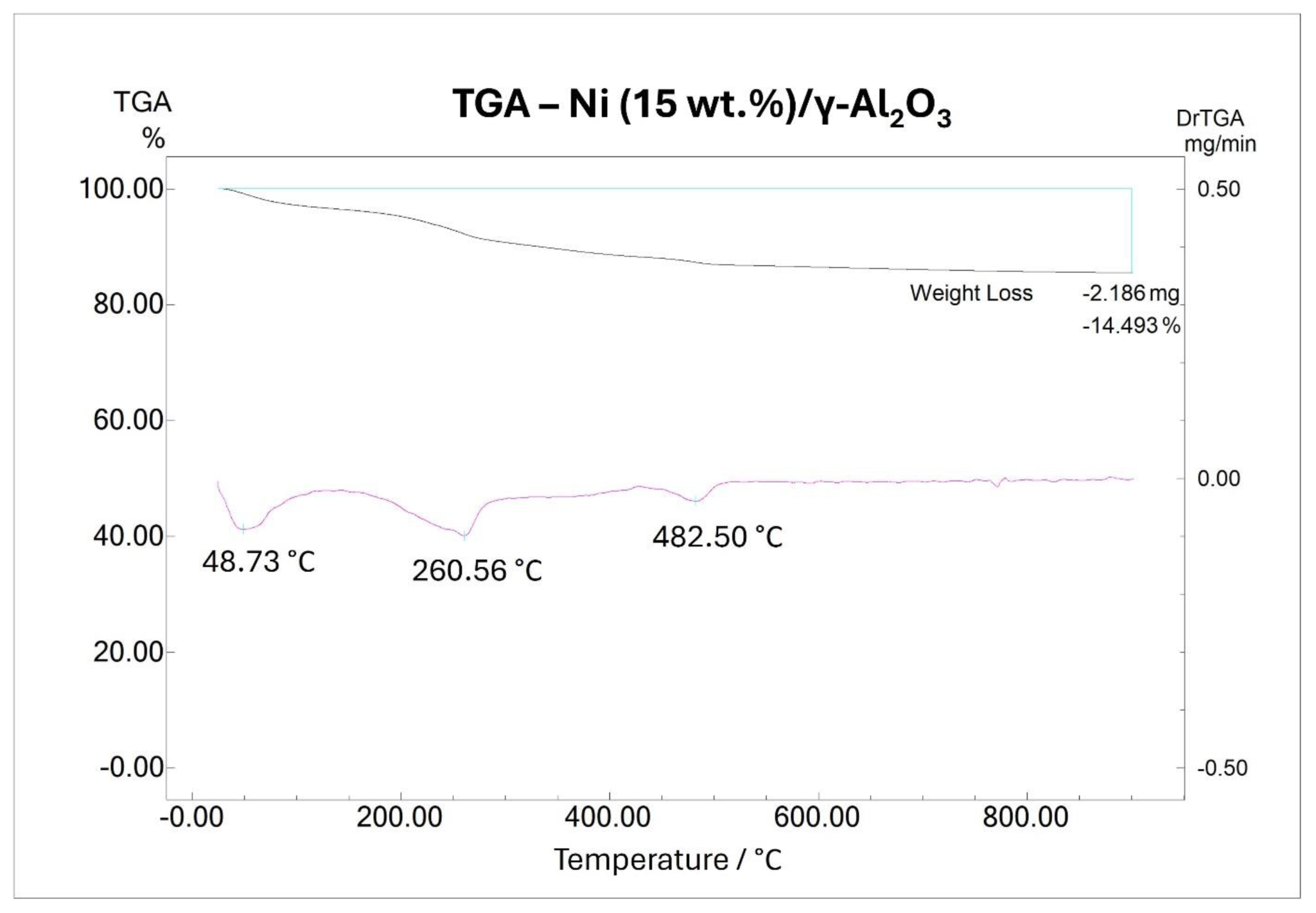

Supplement: Figure S2 — The TGA curve of the Ni(15 wt.%)/γ-Al2O3 catalyst prepared by incipient wetness impregnation method before calcination. [file tjc-48-04-643s2.tif]

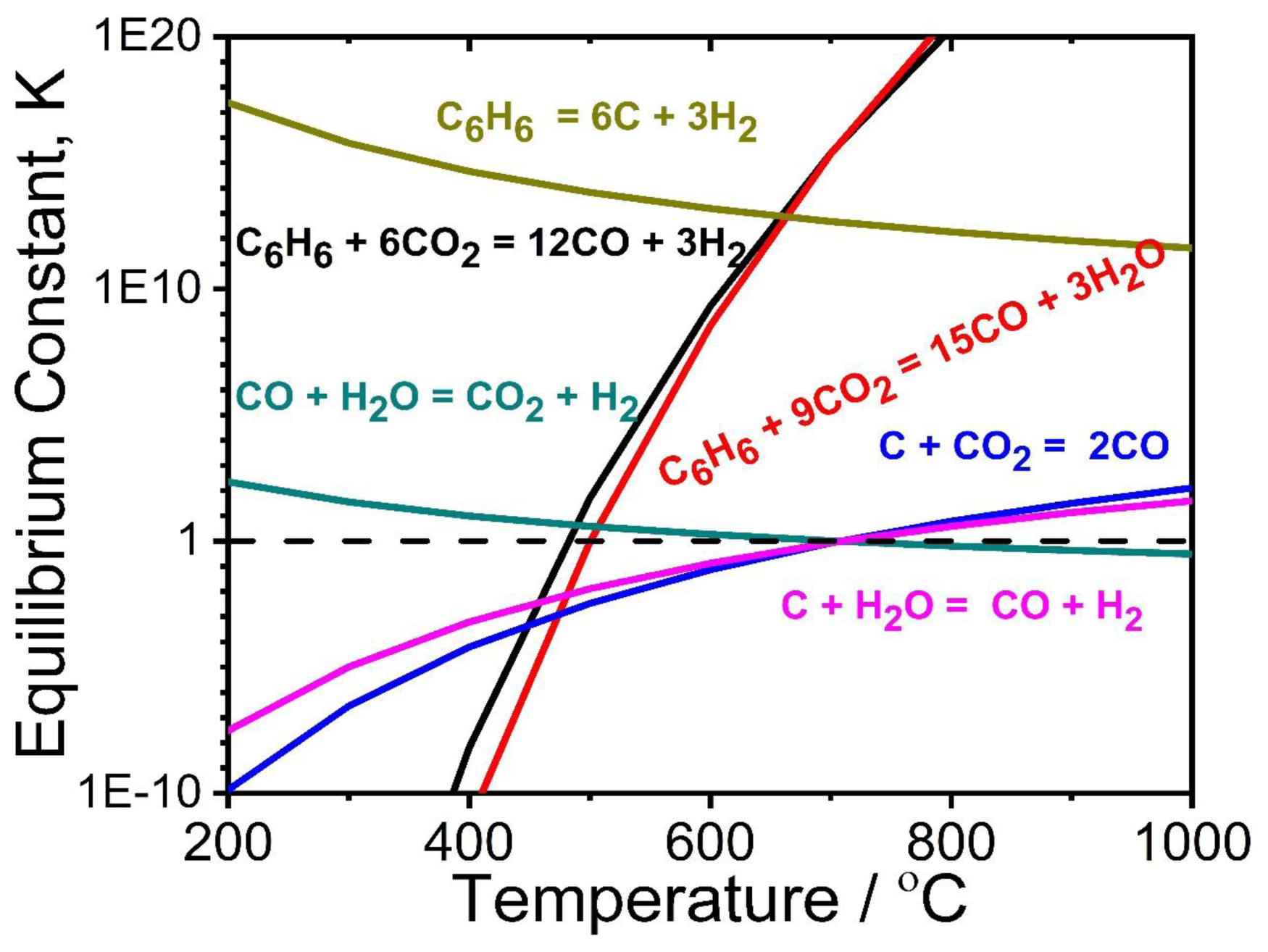

Supplement: Figure S3 — The change of equilibrium constants of reactions occurring at dry reforming conditions as a function of temperature. [file tjc-48-04-643s3.tif]

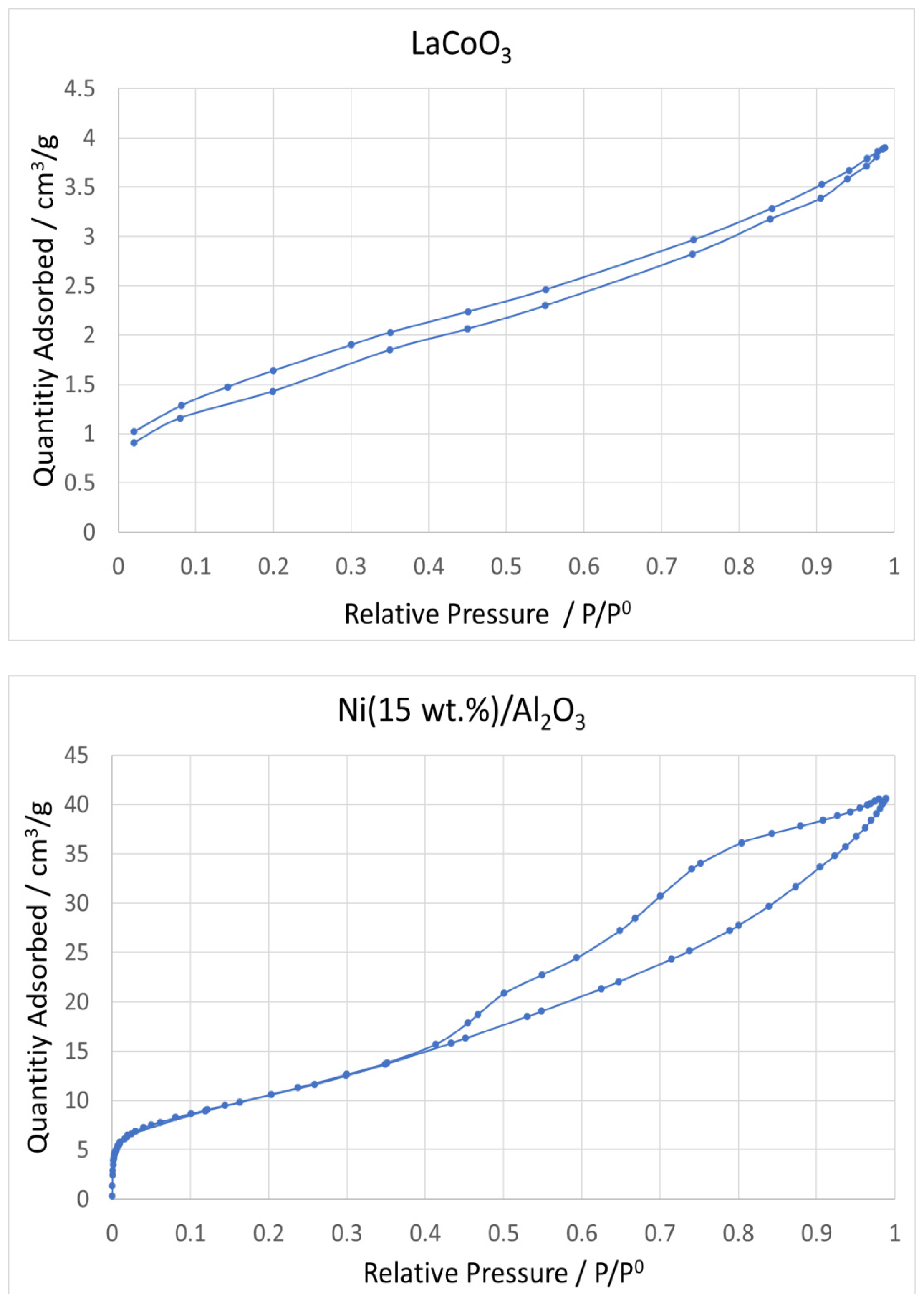

Supplement: Figure S4 — N2 adsorption-desorption isotherms of LaCoO3 (top) and Ni/Al2O3 (bottom). [file tjc-48-04-643s4.tif]

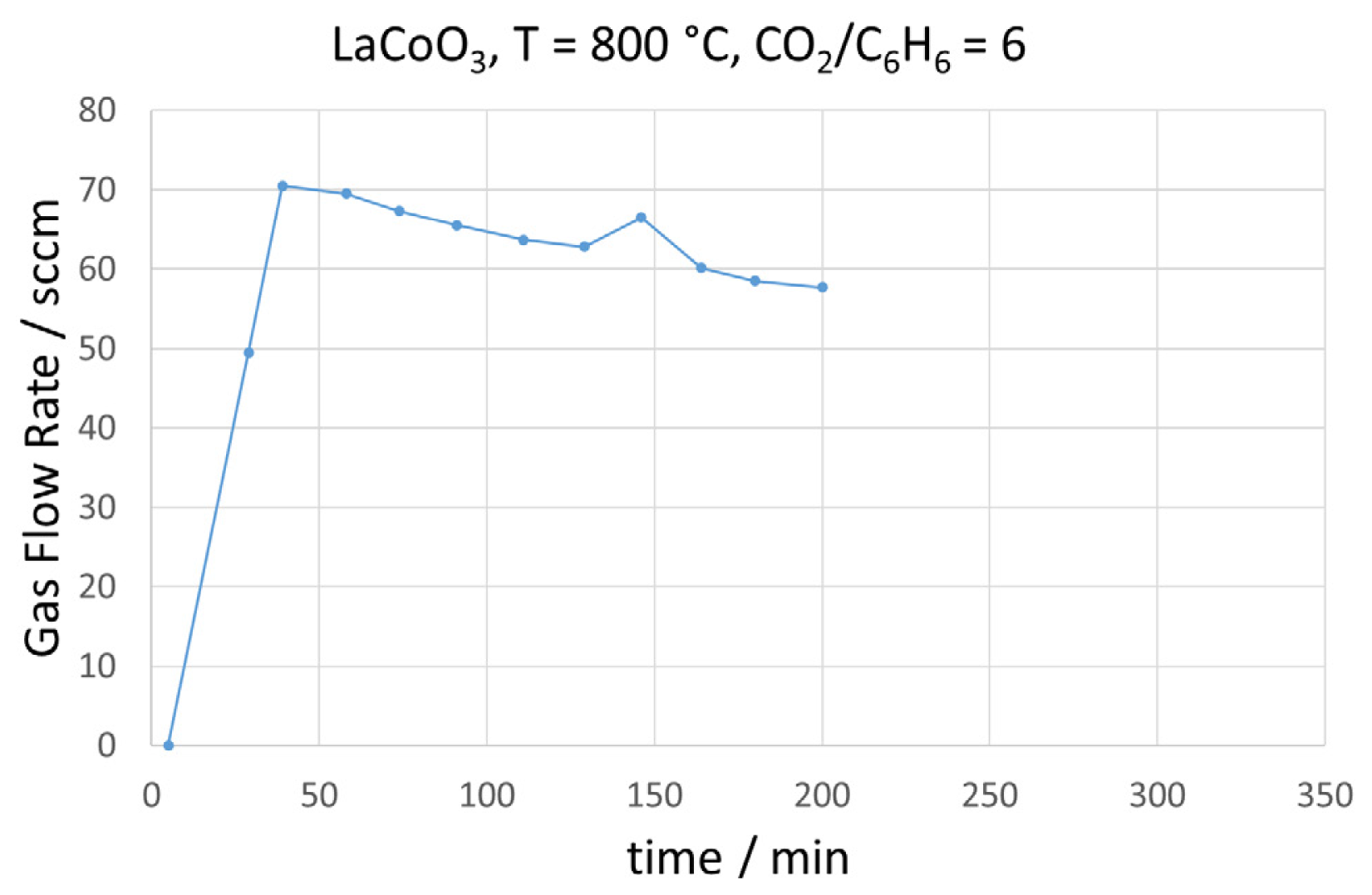

Supplement: Figure S5 — The variation of gas flow rate as function of time for the LaCoO3 catalyst at the conditions of 800 °C and the CO2/C6H6 ratio of 6. [file tjc-48-04-643s5.tif]

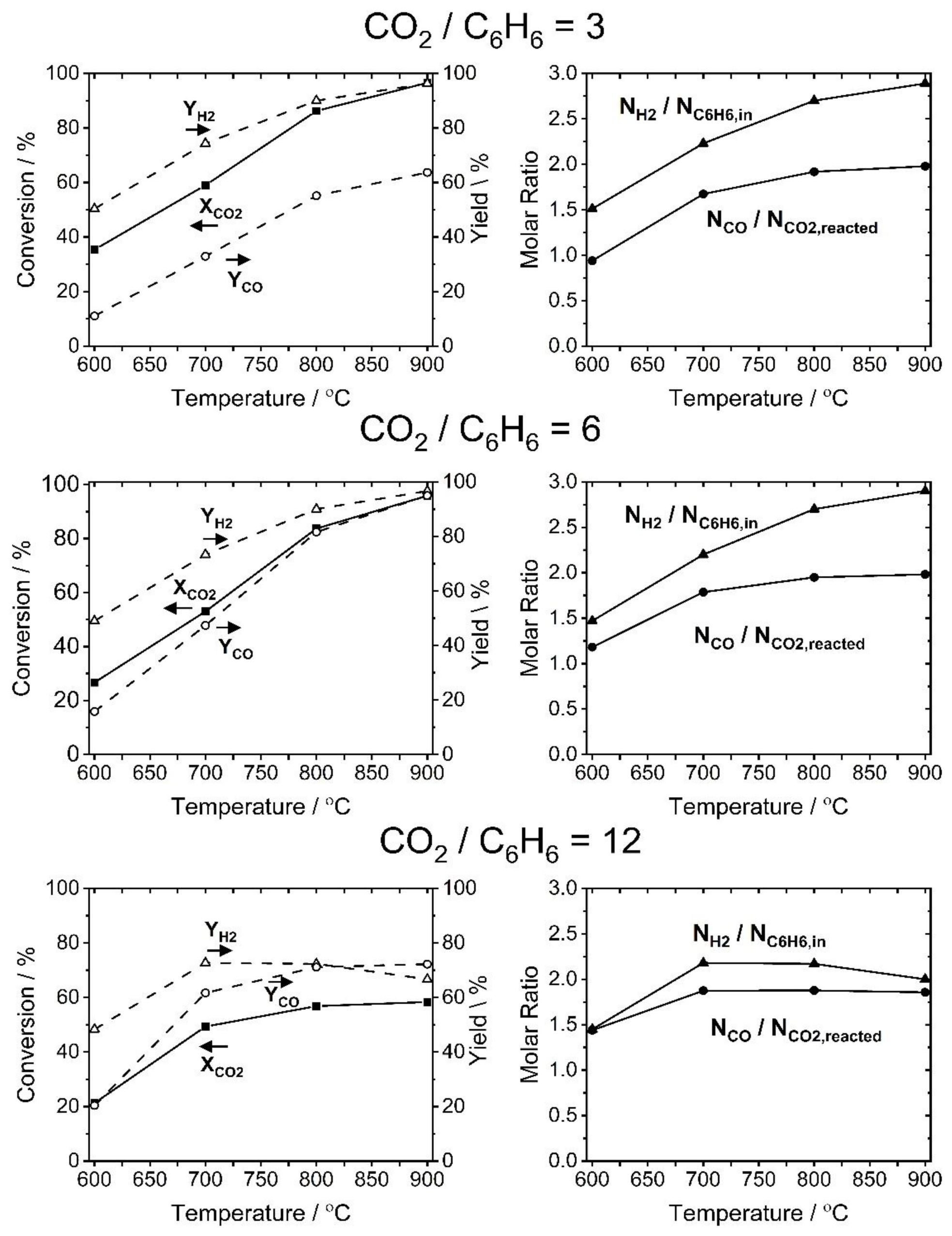

Supplement: Figure S6 — Equilibrium conversion of CO2 and H2 and CO yields (left panel) along with H2/C6H6,in and CO/CO2,reacted ratios at equilibrium conditions (right panel) determined based on Gibbs free energy minimization. [file tjc-48-04-643s6.tif]

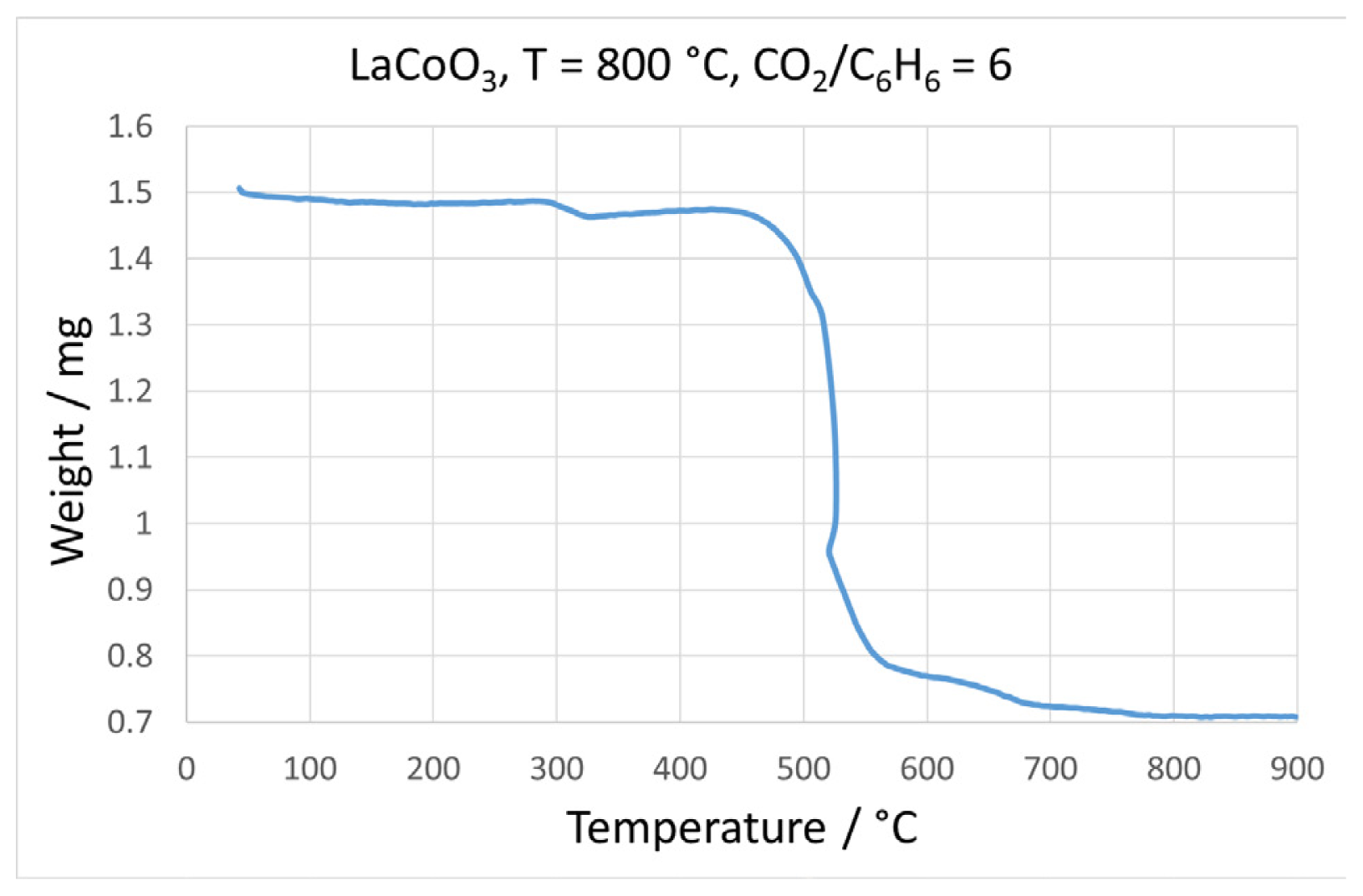

Supplement: Figure S7 — TGA analysis of the used LaCoO3 catalyst obtained after the dry reforming experiment at 800 °C and at the CO2/C6H6 ratio of 6. [file tjc-48-04-643s7.tif]

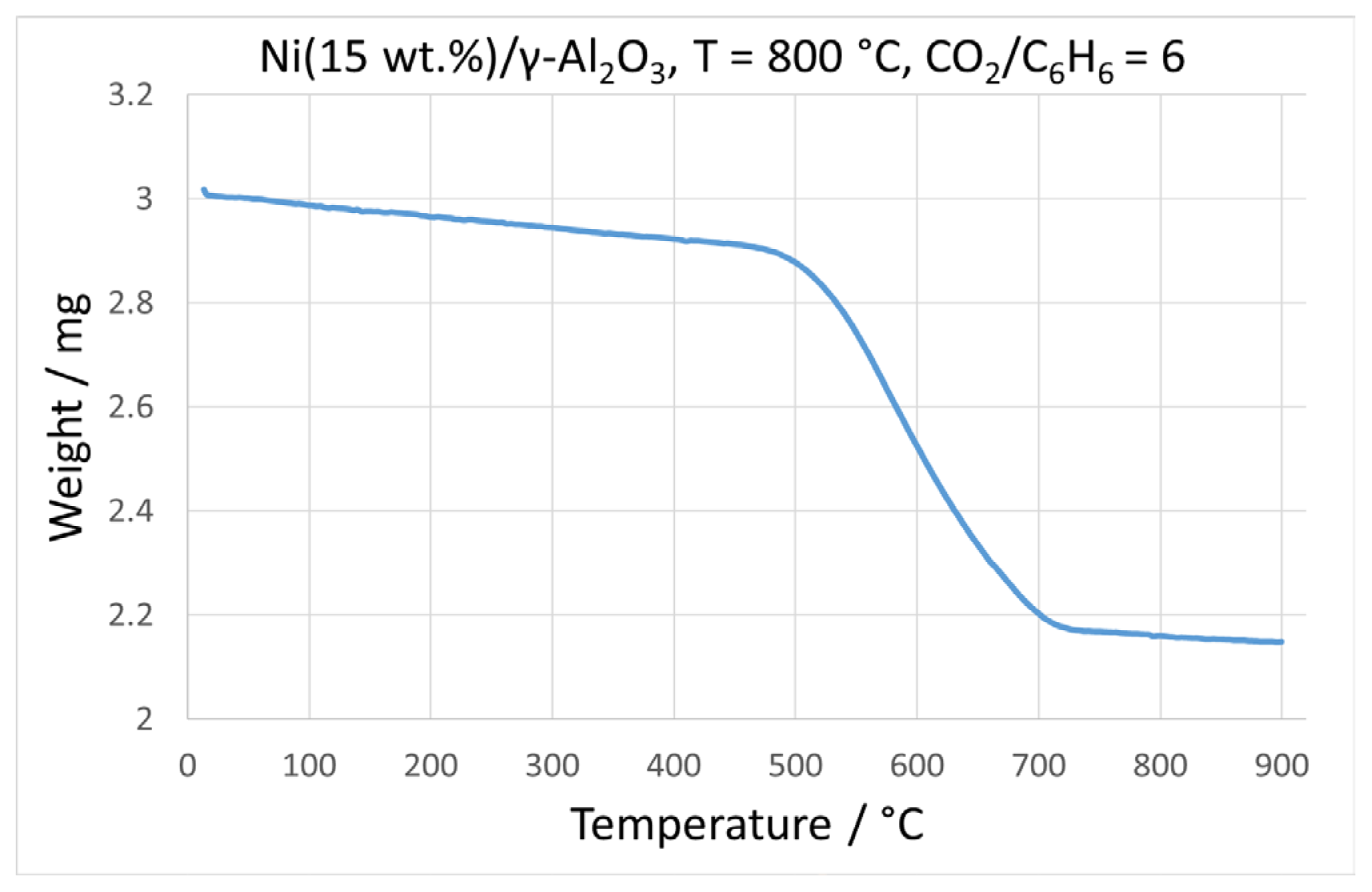

Supplement: Figure S8 — TGA analysis of the used Ni/γ-Al2O3 catalyst obtained after the dry reforming experiment at 800°C and at the CO2/C6H6 ratio of 6. [file tjc-48-04-643s8.tif]

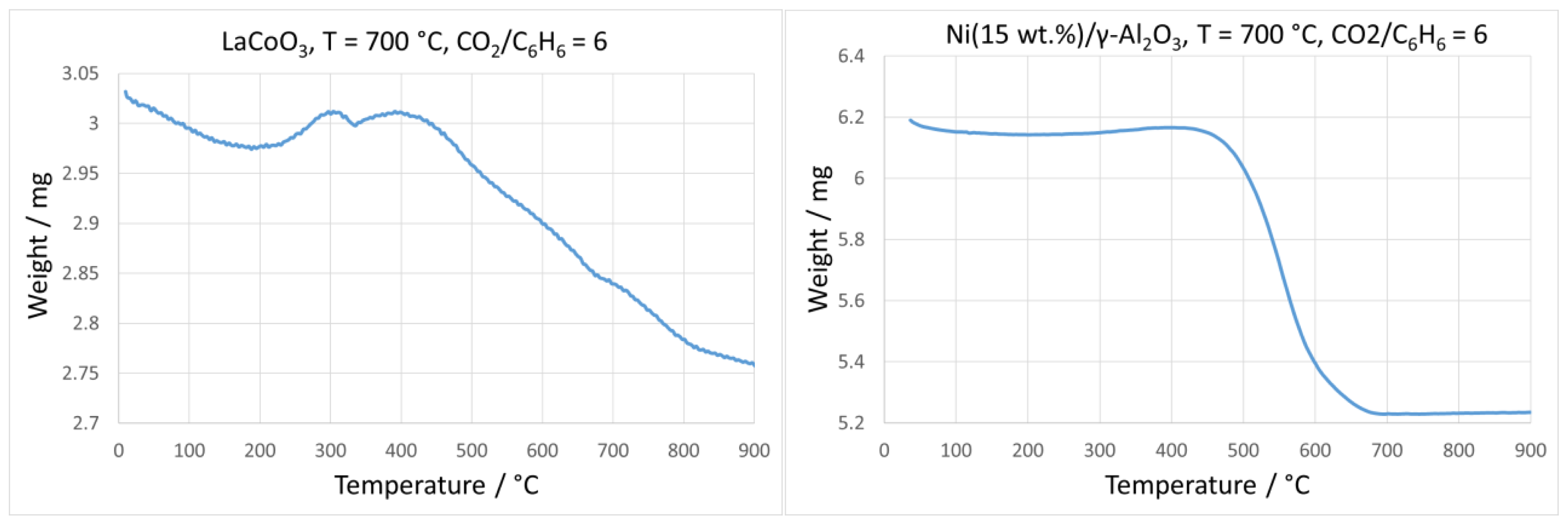

Supplement: Figure S9 — TGA analysis of the used LaCoO3 (left panel) and Ni/γ-Al2O3 (right panel) catalysts obtained after the dry reforming experiment at 700 °C and at the CO2/C6H6 ratio of 6. [file tjc-48-04-643s9.tif]
